# Process Parameter Optimization for CO_2_ Laser Polishing of Fused Silica Using the Taguchi Method

**DOI:** 10.3390/ma17030709

**Published:** 2024-02-01

**Authors:** Guanghua Lu, Xiaopeng Li, Dasen Wang, Kehong Wang

**Affiliations:** 1 School of Mechanical and Engineering, Nanjing University of Science and Technology, Nanjing 210094, China; 2 School of Material Science and Engineering, Nanjing University of Science and Technology, Nanjing 210094, China

**Keywords:** CO_2_ laser polishing, Taguchi design, fused silica glass, process parameters, roughness

## Abstract

Fused silica was polished to a high quality by a CO_2_ laser beam with a rapid scanning rate. The rapid scanning rate produced a line laser heat source, resulting in a “polishing line” during the polishing process. The Taguchi method was used to evaluate the comprehensive influence of polishing process parameters on the polishing qualities. Four factors, namely the length of laser reciprocating scanning (A), laser beam scanning speed (B), feed speed (C), and defocusing amount (D), were investigated in this study. The optimal process parameter combination (A1B1C1D1) was obtained. The surface roughness of fused silica was reduced from Ra = 0.157 μm to 0.005 μm. Through analysis of variance (ANOVA), it was found that laser beam scanning speed (B) had a significant influence on the polishing quality. The interaction of the two factors plays a decisive role in the determination of the best process parameters, and the influence of other multi-factor interaction can be ignored; the interaction between A × B is the largest, with a contribution of 42.69%.

## 1. Introduction

Fused silica is the amorphous state of SiO_2_ [1,2]. It has been used as an optical element in the high-power laser equipment field because of its good physical, chemical, optical, and thermodynamic properties [2,3]. In the traditional mechanical polishing process, scratches, microcracks, impurities, and other defects are produced at the surface and subsurface of optical components, leading to damage under strong laser irradiation [4,5]. Laser polishing, which is characterized as a non-contact polishing technology has none of the defects associated with traditional mechanical polishing. It can also repair the microcracks caused by the grinding and improve the surface properties of the polished parts, and it can be used to make micro-optical elements such as lenticular lens arrays and microlens arrays [6,7]. Due to the interaction of glass material and laser radiation, a thin surface layer of the glass is heated up just below evaporation temperature. Increasing temperature results in reduced viscosity in the surface layer. Due to the surface tension, the profile peaks are leveled, and the valleys are filled, leading to the reduction of the roughness. Compared to traditional polishing, laser polishing has the advantage of reducing the microroughness, with spatial wavelength λ < 100 μm [6].

Many researchers have used CO_2_ lasers to polish fused silica glass, as fused silica has strong absorption capability with regard to the CO_2_ laser. For instance, P.A. Temple introduced two surface treatment schemes: one was the single-channel CO_2_ laser polishing process (the part scan rate was ~5 mm/s), and the other was the multi-channel CO_2_ laser polishing process (the beam traversed at 8 mm/s). It was found that the laser damage resistance of the surface of fused silica glass treated by laser irradiation was improved, but due to the use of the Gaussian CW laser and slow scanning speed, the polishing caused residual stress in the substrate, even cracking in serious cases, with low processing efficiency [8]. To improve the polishing efficiency, the active beam integration was achieved by multi-faceted mirror vibration, enabling scanning of an area of 80 mm^2^. This method belongs to the early galvanometer scanning polishing [9]. In order to reduce the thermal impact caused by continuous polishing and improve the stability of processing, an acoustic-optic modulator (AOM) was incorporated into the optical path, and the non-planar optical elements were polished [10,11]. In addition, the optical components were polished by controlling the movement of the mobile platform (X–Y), and the pulse width of 500 μs was converted into a preheating pulse and a series of adjustable duty factor micro-pulses through the acoustic-optic modulator, which reduced the instability of output power and maintained a high surface temperature without exceeding 2700 °C, avoiding undesired material ablation during polishing. The silica surface roughness was smoothed from ~1 μm scale down to levels < 1 nm, and the polishing efficiency was 16 cm^2^/min [12]. However, the pulse polishing efficiency of the laser polishing process is low due to its slow scanning speed. To improve the polishing efficiency, H. Kerstin produced a focus line (also known as a “polishing line”) with a continuous laser by scanning the laser with a speed of 400–800 mm/s. With the “polishing line” moving with a slow feed speed (≤50 mm/min), a homogenous temperature distribution in the “polishing line” was achieved, and the surface of fused silica glass was polished [1,6,13]. Furthermore, a laser beam scanning rate up to 10,000 mm/s was also employed to further improve the polishing efficiency, and the roughness of fused silica surfaces were reduced, from Ra = 100 nm to Ra < 6 nm, with the polishing efficiency reaching up to 1 cm^2^/s [14,15].

At present, research is mainly focused on the influence of individual factors (such as power, scanning speed, defocus amount, etc.) on polishing quality, rarely considering the interaction between various factors and especially lacking in the interaction between three and even more factor interactions. The challenge of laser polishing is that several parameters must be adjusted to achieve a perfect glass surface, which largely depend on the incident energy and its stability. When the energy accumulated by laser irradiation and heat during the polishing process causes the surface temperature of the glass to be slightly lower than the evaporation temperature, the surface of the fused quartz is fully melted, and the surface quality is perfect. However, the comprehensive influence of polishing process parameters on the polishing qualities needs further research. The length of the “polishing line”, the energy density, the laser scanning speed, and the feed rate of the “polishing line” are the main factors affecting the temperature of the polishing zone. Due to the difficulty in accurately measuring the length of the “polishing line”, this article took the length of laser reciprocating scanning as a research factor. The change in defocus amount would change the size of the light spot, thereby affecting the magnitude of energy density. Four factors, namely the length of laser reciprocation (A), laser beam scanning speed (B), feed speed (C), and defocusing amount (D), with different levels, were investigated using the Taguchi method in this study. The influence of different factors and their interactions on the surface roughness of polished surfaces are discussed.

## 2. Experimental Design

**Figure 1:**
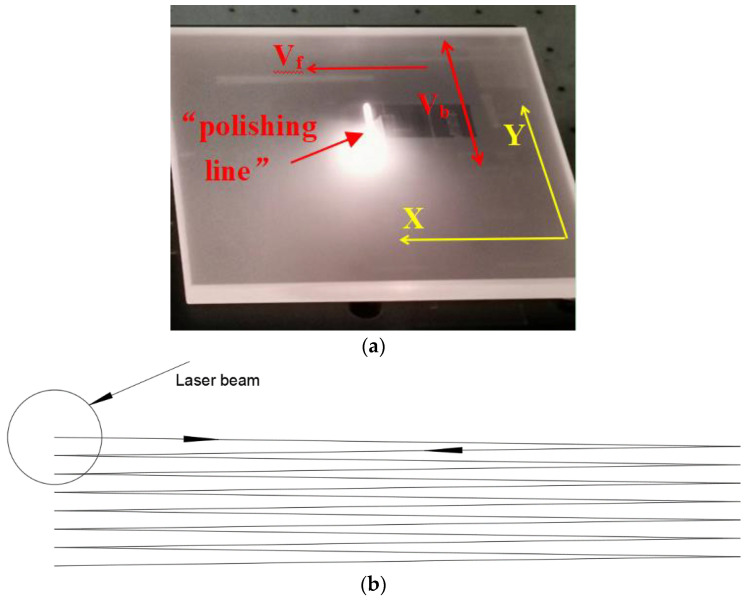
Fused silica glass sample during the polishing process and scan trajectory. (**a**) Fused silica glass sample during the polishing process; (**b**) scan trajectory.

A CW CO_2_ laser at 10.6 μm wavelength, with a maximum power of 120 W, was used for laser polishing. Figure 1 shows the fused silica sample and scanning trajectory during polishing. Figure 1a) shows the laser beam forms a “polishing line” instead of a “polishing point” with a high-speed V_b_ in one axis (Y), and the sample moves in the direction of the other axis (X) at a few millimeters per second (V_f_) to achieve area polishing. Figure 1b) shows the scan trajectory of laser beam.

The orthogonal trials of the four factors were carried out three times on fused silica glass with a size of 50 × 50 × 3 mm^3^. The selected process parameters and their levels are presented in Table 1. The L_16_ (2^15^) Orthogonal Array was selected for conducting experiments. Each group of experiments was repeated three times; then, the average value was taken to reduce the influence of errors on the experiment results. A KEYENCE confocal laser scanning microscope (measuring range: 107 μm × 143 μm) was used to observe the surface roughness of the samples. The grinding surface roughness of the three samples was Ra = 0.157 μm, Ra = 0.180 μm, and Ra = 0.187 μm.

## 3. Results and Discussion

### 3.1. Signal-to-Noise Ratio Analysis

In order to study the influence of factors and their interaction on the quality of CO_2_ laser polishing, the interactions were considered in the design of the mater head. The orthogonal array experimental layout is detailed in Table 2.

There are controllable factors and uncontrollable factors in the experiments. In radio communication, the ratio of signal power to noise power is expressed as the signal-to-noise (S/N) ratio. The error in the test is equivalent to the noise in radio communication. By analyzing the test data through the S/N ratio, the influence of uncontrollable factors can be avoided, and the results of multiple repeated tests (raw data) can be combined into a single number [16]. The S/N ratio reflects the dispersion characteristics and average characteristics of the test results; it is used to judge the stability of the test, which is a comprehensive index [17]. In this paper, the S/N ratio is used in the Taguchi method to analyze the relationship between the main effect and the error effect in the polishing quality. According to the different quality characteristics, S/N ratio analysis can be divided into “the more nominal the better”, “the lower the better”, “the larger the better”.

This study aims to minimize the roughness of the laser polishing surface; thus, “the lower the better” quality characteristic is considered. Equation (1) is used to find the S/N ratio for the “lower is better” quality characteristics:

(1) $$S/N=-10lg\left(\frac{1}{n}\sum_{i=1}^{n}y_{i}^{2}\right)$$

where y_i_ (i = 1,2, …, n) is the response value of the ith test condition, and n is the total number of trials.

Table 3 shows the raw data of test roughness and the corresponding S/N ratio. Range analysis was used to evaluate the influence of each factor on the test results, and the results are shown in Table 4. K_i_ (i = 1, 2) represents the sum of the roughness S/N ratio results obtained by different factors of level i (i = 1, 2), and k_i_ (i = 1, 2) represents the average roughness S/N ratio results under different factors at level i (i = 1, 2). For example, K_1_ and k_1_, with a length of 6 mm for the length of laser reciprocation (A), are calculated as follows:

(2) $$K_{1}=\sum_{i=1}^{8}y_{i}=y_{1}+y_{2}+y_{3}+y_{4}+y_{5}+y_{6}+y_{7}=162.977$$

(3) $$\bar{A_{1}}=k_{1}=\frac{K_{1}}{8}=20.372$$

**Figure 2:**
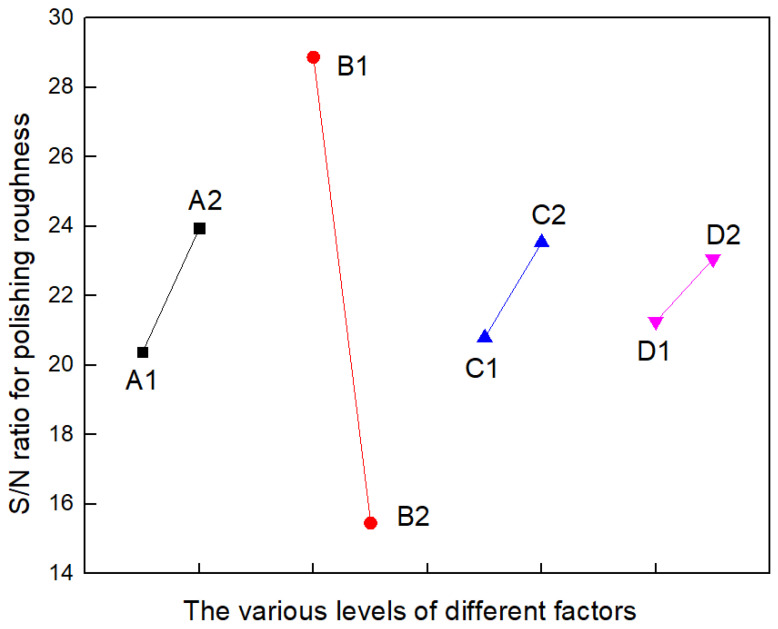
Orthogonal experimental factor effect trend of CO_2_ laser polishing.

**Figure 3:**
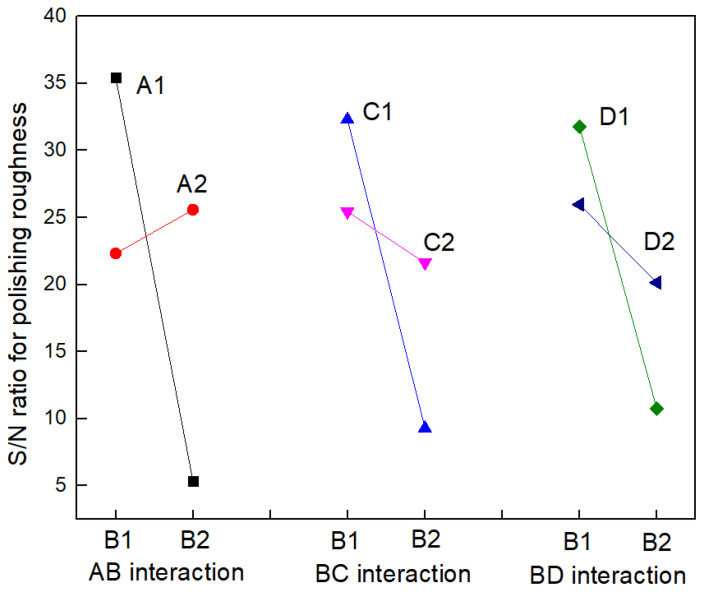
Orthogonal two-factor interaction trend.

In Table 4, the k_i_ maximum is the optimal level parameter for the process, and the optimal level combination is A2B1C2D2. The order of influence of factors on the index according to the size of the extreme value of R is AB > B > BC > BD > A > C > ACD > ABCD > D > AC > ABD > CD > AD > ABC > BCD. A trend chart of factor effects can be plotted by analyzing the experimental results (Table 4) and the different levels of effects of each factor. As shown in Figure 2, laser beam scanning speed (B) plays a major role, and other factors can be ignored. It can be seen from the extreme R value that among the factor interaction, AB, BC, and BD have the greatest impact, and from the results (Table 4) that the valuation and interaction trend of different levels of the interaction of three factors—AB, BC, and BD—can be obtained, which are shown in Table 5 and Figure 3. It can be seen from Figure 3 that the two lines in the interaction diagram of AB, BC, and BD cross each other, indicating that the interaction between the two factors does exist and cannot be ignored, and the interaction between AB is the largest. When the length of laser reciprocation (A) is 6 mm, the S/N ratio decreases sharply as the laser beam scanning speed (B) decreases. When the length of laser reciprocation (A) is 7 mm, the S/N ratio slowly increases as the laser beam scanning speed (B) decreases. When feed speed (C) is 1.5 mm/s, the S/N ratio decreases rapidly as the laser beam scanning speed (B) decreases. When feed speed (C) is 2 mm/s, the S/N ratio slowly decreases as the laser beam scanning speed (B) decreases. When the defocusing amount (D) is +1 mm, the S/N ratio decreases rapidly as the laser beam scanning speed (B) decreases. When the defocusing amount (D) is +5 mm, the S/N ratio slowly decreases as the laser beam scanning speed (B) decreases.

**Figure 4:**
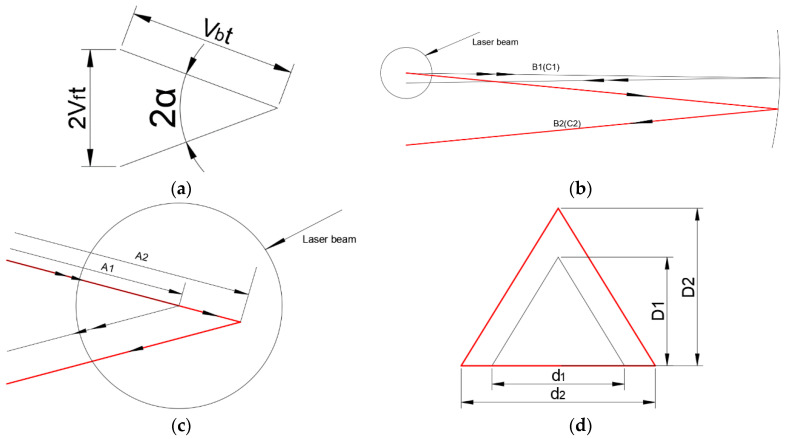
Schematic diagram of the influence of each factor on energy (double arrows and thick red solid lines indicate after change, single arrows and black thin solid lines indicate before change, and thick solid lines are displayed when they overlap). (**a**) The relationship between α and V_b_. (**b**) The change in laser beam scanning speed (B) and V_f_ feed speed (C). (**c**) The change in length of the laser reciprocation (A). (**d**) The change in defocusing amount (D).

It can be seen from Figure 4a that sinα = V_f_t/(V_b_t) = V_f_/V_b._ Under the condition that other process parameters remain unchanged, the increase of the feed speed V_f_ or the decrease of the laser beam scanning speed V_b_ will lead to an increase in the angle α between the trajectory, thereby reducing the overlap ratio and thermal accumulation between the trajectory (X axis direction in Figure 2) and leading to a decrease in the temperature of the polishing area; the flow time of the molten pool is also shortened [14]. As shown in Figure 4b, laser beam scanning speed (B) not only affects the duration of interaction time between laser radiation and glass (i.e., the duration of continuous laser irradiation at a certain point) but also affects the overlap ratio and thermal accumulation between the trajectory. As laser beam scanning speed (B) increases, the irradiation time at a certain point decreases, and the temperature in the polishing area decreases. But an increase in laser beam scanning speed (B) will result in an decrease of the angle α between the trajectory and an increase the overlap ratio and thermal accumulation between the trajectory, resulting in an increase in the temperature of the polishing zone. As analyzed earlier, laser beam scanning speed (B) has a greater impact on roughness than feed rate (C). Therefore, it can be inferred that an increase in laser beam scanning speed (B) will cause an increase in the temperature of the polishing zone, which is consistent with the experimental results. On the contrary, a decrease in laser beam scanning speed (B) will lead to a decrease in the temperature of the polishing zone. Feed rate (C) affects the thermal accumulation between the trajectory. Increasing laser beam scanning speed (B) will reduce the overlap ratio and thermal accumulation between the trajectory, leading to an increase in the temperature of the polishing zone. Reducing laser beam scanning speed (B) will increase the overlap ratio and thermal accumulation between the trajectory, leading to a decrease in the temperature of the polishing zone. As shown in Figure 4c, the length of laser reciprocation (A) influences the interaction time between the trajectory lines of laser radiation and glass. Under the condition that other process parameters remain unchanged, the length of laser reciprocating increases, the angle α between the trajectory is constant, the overlap ratio and thermal accumulation between the trajectory decreases, the temperature of the polishing area decreases, and the flow time of the molten pool is shortened. As shown in Figure 4d, the defocusing amount (D) affects the size of the spot, and under the condition that other process parameters remain unchanged, as the defocusing amount increases, the energy density in the spot decreases, and the temperature of the polishing area decreases accordingly.

**Figure 5:**
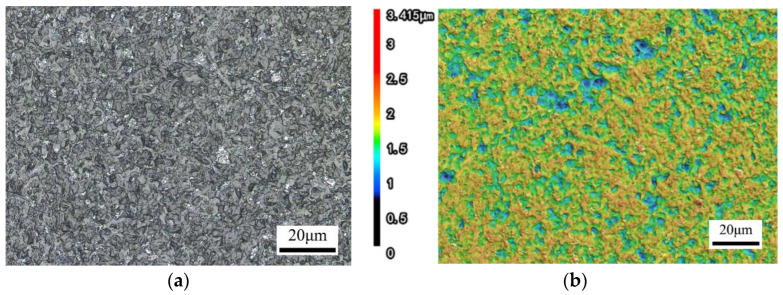
Surface morphology after grinding of fused silica glass (Ra = 0.157 μm). (**a**) 2D; (**b**) 3D.

**Figure 6:**
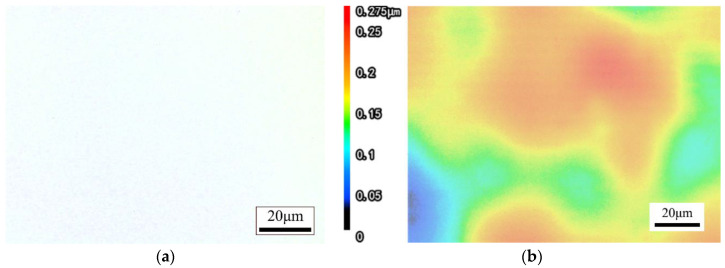
Surface morphology after CO_2_ laser polishing of fused silica glass (Ra = 0.005 μm). (**a**) 2D; (**b**) 3D.

It can be seen from the order of extreme value R in Table 4 that the influence of the interaction between the length of laser reciprocation (A) and laser beam scanning speed (B) is greater than that of the laser beam scanning speed (B), and the selection of laser beam scanning speed (B) must take into account the combination of the length of laser reciprocation (A) and laser beam scanning speed (B). Thus, the optimal level of combination depends on the result of two-factor interaction optimization. It can be seen from Table 5 and Figure 3 that the optimal combination between the two factors is A1B1, B1C1, and B1D1; thus, the optimal level after optimization is A1B1C1D1, that is, the length of laser reciprocating (A) is 6 mm, the laser beam scanning speed (B) is 4500 mm/s, the feed speed (C) is 1.5 mm/s, and the defocusing amount (D) is +1 mm. This parameter combination resulted in the lowest roughness value Ra = 0.005 μm in 16 experiments, so the optimized process combination considering the interaction effect is correct. The surface morphology of fused silica glass before and after polishing is shown in Figure 5 and Figure 6. To quantify the roughness Ra of a laser polished surface, four measurements with different magnifications are made with a KEYENCE confocal laser scanning microscope. By changing the sampling length, the roughness under different measurement areas was measured, and the results are shown in Table 6.

### 3.2. Analysis of Variance (ANOVA)

**Figure 7:**
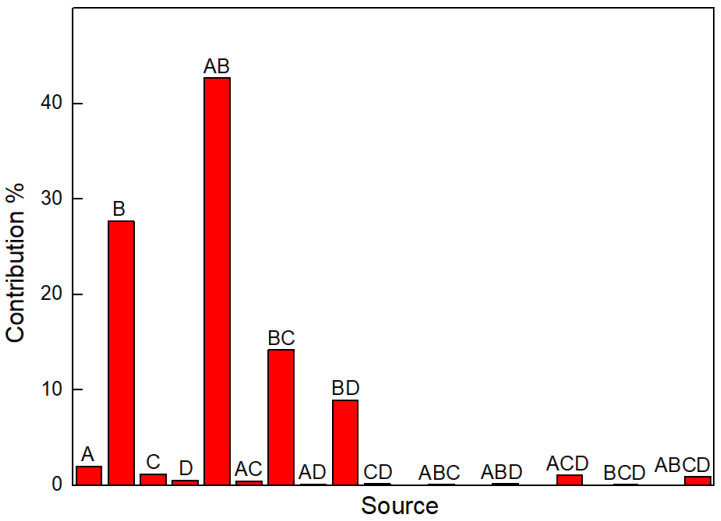
The contribution of different factors and their interaction to the S/N ratio of surface roughness.

ANOVA is a statistical method that is used to determine the interactions of the process parameters used in the test design [18]. An analysis of variance was carried out to study the relative significance of control factors and their contribution to the performance characteristic, i.e., the roughness of the laser polishing surface. The ANOVA results for roughness are shown in Table 6 and Figure 7. The ratio of the mean of the squared deviations to the mean of the squared error is the F value for each parameter.

It can be seen from Table 7 and Figure 7 that factor A has a certain influence and factor B has a particularly significant effect. The interaction of AB, BC, and BD have a particularly significant influence, among which the interaction of AB contributes the most, at 42.69%. Other factors and interactions are insignificant.

## 4. Conclusions

In this paper, the relationship between process parameters and their interaction on the roughness of CO_2_ laser polishing was clarified using S/N ratio analysis and ANOVA. Different from the traditional study of factor effects, the factors and their interaction were studied in this paper. The main results are summarized as follows:

1. The influence of interaction is considered in CO_2_ laser polishing of fused silica. Although factor B has a certain influence, the interaction of two factors determines the selection of the optimal level in combination.
2. The temperature of the polishing area and the flow time of the molten pool are important factors affecting the surface roughness after polishing, and on the basis of studying the influence of various factors and their interaction on the roughness of the polished surface, the law of the influence of each factor on the interaction time between laser radiation and glass and the flow time of the molten pool is revealed.
3. The interaction is considered in the variance analysis, and the optimal process combination A1B1C1D1 is optimized on the basis of range analysis through binary tables and graphs; the roughness of the fused silica grinding surface is reduced from Ra = 0.157 μm to 0.005 μm. The effective reduction rate of roughness value is as high as 96.8%.
4. In the laser polishing process, including laser reciprocating scanning and the sample moving slowly on the platform, the interaction of two-factor AB, BC, and BD has a highly significant impact, which is recorded as “***”. Among them, the interaction of AB is the largest, with a contribution of 42.69%; the impact of the other two- factor interactions can be ignored. Factor B has a certain influence, which is recorded as “*”, with a contribution of 27.64%, and its factors can be ignored. The effect of multivariate interaction is negligible.

## Figures and Tables

**Table 1:** Parameters of orthogonal test.

Factors	Unit	Levels	
		1	2
A: Length of laser reciprocation	mm	6	7
B: Laser beam scanning speed	mm/s	4500	1500
C: Feed speed	mm/s	1.5	2
D: Defocusing amount	mm	1	5

**Table 2:** Orthogonal array experimental layout.

Exp. No.	Column Number														
	1	2	3	4	5	6	7	8	9	10	11	12	13	14	15
	A	B	AB	C	AC	BC	ABC	D	AD	BD	ABD	CD	ACD	BCD	ABCD
1	1	1	1	1	1	1	1	1	1	1	1	1	1	1	1
2	1	1	1	1	1	1	1	2	2	2	2	2	2	2	2
3	1	1	1	2	2	2	2	1	1	1	1	2	2	2	2
4	1	1	1	2	2	2	2	2	2	2	2	1	1	1	1
5	1	2	2	1	1	2	2	1	1	2	2	1	1	2	2
6	1	2	2	1	1	2	2	2	2	1	1	2	2	1	1
7	1	2	2	2	2	1	1	1	1	2	2	2	2	1	1
8	1	2	2	2	2	1	1	2	2	1	1	1	1	2	2
9	2	1	2	1	2	1	2	1	2	1	2	1	2	1	2
10	2	1	2	1	2	1	2	2	1	2	1	2	1	2	1
11	2	1	2	2	1	2	1	1	2	1	2	2	1	2	1
12	2	1	2	2	1	2	1	2	1	2	1	1	2	1	2
13	2	2	1	1	2	2	1	1	2	2	1	1	2	2	1
14	2	2	1	1	2	2	1	2	1	1	2	2	1	1	2
15	2	2	1	2	1	1	2	1	2	2	1	2	1	1	2
16	2	2	1	2	1	1	2	2	1	1	2	1	2	2	1

**Table 3:** Raw data of test roughness and the corresponding S/N ratio.

Exp. No.	A	B	C	D	Raw Data of Roughness/μm			S/N Ratio for Roughness
					S1	S2	S3	
1	1	1	1	1	0.005	0.006	0.007	44.357
2	1	1	1	2	0.012	0.017	0.019	35.773
3	1	1	2	1	0.015	0.020	0.023	34.149
4	1	1	2	2	0.044	0.038	0.046	27.371
5	1	2	1	1	0.956	1.314	1.472	−2.048
6	1	2	1	2	0.577	0.865	1.068	1.304
7	1	2	2	1	0.397	0.758	0.889	2.946
8	1	2	2	2	0.161	0.094	0.044	19.125
9	2	1	1	1	0.027	0.048	0.057	26.790
10	2	1	1	2	0.069	0.079	0.082	22.285
11	2	1	2	1	0.063	0.079	0.099	21.759
12	2	1	2	2	0.109	0.110	0.138	18.434
13	2	2	1	1	0.130	0.198	0.335	12.510
14	2	2	1	2	0.088	0.022	0.023	25.348
15	2	2	2	1	0.044	0.026	0.026	29.602
16	2	2	2	2	0.016	0.021	0.017	34.832

**Table 4:** Range analysis results of S/N ratio.

	A	B	AB	C	AC	BC	ABC	D	AD	BD	ABD	CD	ACD	BCD	ABCD
	1	2	3	4	5	6	7	8	9	10	11	12	13	14	15
K_1_	162.977	230.92	243.944	166.32	184.016	215.712	180.256	170.064	180.304	207.664	181.768	181.368	187.8	176.152	167.36
K_2_	191.56	123.616	110.592	188.216	170.528	138.824	174.288	184.472	174.232	146.872	172.768	173.168	166.736	178.384	187.176
k_1_	20.372	28.865	30.493	20.790	23.002	26.964	22.532	21.258	22.538	25.958	22.721	22.671	23.475	22.019	20.920
k_2_	23.945	15.452	13.824	23.527	21.316	17.353	21.786	23.059	21.779	18.359	21.596	21.646	20.842	22.298	23.397
R	3.573	13.413	16.669	2.737	1.686	9.611	0.746	1.801	0.759	7.599	1.125	1.025	2.633	0.279	2.477

**Table 5:** Two-factor interaction analysis of orthogonal test.

Interaction between Factor A and Factor B				
	A1	A2	∑	Average
B1	35.413	22.317	57.73	28.865
B2	5.332	25.573	30.905	15.453
∑	40.745	47.89	93.159	
Average	20.373	23.945		22.159
Interaction between factor B and factor C				
	C1	C2	∑	Average
B1	32.301	25.428	57.729	28.865
B2	9.279	21.626	30.905	15.453
∑	41.58	47.054	88.634	
Average	20.79	23.527		22.159
Interaction between factor B and factor D				
	D1	D2	∑	Average
B1	31.764	25.966	57.73	28.865
B2	10.753	20.152	30.905	15.453
∑	42.517	46.118	88.635	
Average	21.259	23.059		22.159

**Table 6:** Measurement area for determining roughness.

Measurement Area (μm^2^)	Initial (μm)	Resolution (μm)	Magnification
143 × 107	0.157	0.005	100×
274 × 206	0.191	0.01	50×
696 × 522	0.736	0.041	20×
1392 × 1044	1.692	0.135	10×

**Table 7:** Analysis of variance for S/N ratio of surface roughness.

Source	Sum of Variance	df	Mean Value	F	Contribution
A	51.021	1	51.021	4.218 *	1.96%
B	719.59	1	719.59	59.495 ***	27.64%
C	29.92	1	29.92		1.15%
D	12.93	1	12.93		0.5%
A × B	1111.378	1	1111.378	91.887 ***	42.69%
A × C	11.681	1	11.681		0.45%
B × C	369.441	1	369.441	30.545 ***	14.19%
A × D	2.26	1	2.26		0.09%
B × D	230.935	1	230.935	19.093 ***	8.87%
C × D	4.158	1	4.158		0.16%
A × B × C	2.536	1	2.536		0.1%
A × B × D	5.018	1	5.018		0.19%
A × C × D	27.686	1	27.686		1.06%
B × C × D	0.267	1	0.267		0.01%
A × B × C × D	24.498	1	24.498		0.94%
Error	120.954	10	12.095		
Total	2603.319	15			
Critical value F_α_: F_0.01_(1, 10) = 10.04, F_0.05_(1, 10) = 4.96, F_0.1_(1, 10) = 3.29					

Note: When F > F_0.01_, the influence of factors is particularly significant, and it is recorded as “***”. When F_0.01_ ≥ F > F_0.05_, the influence of factors is significant, and it is recorded as “**”. When F_0.05_ ≥ F > F_0.1_, there is a certain impact, which is recorded as “*”. When F_0.1_ ≥ F, there is little or no effect.

## Data Availability

The raw data generated during the present study are available from the corresponding author on reasonable request.
